# Image versus Health: The Role of Perceptions of Masculinity on Sexual Risk Behaviors among HIV-Positive African American Men who have Sex with Men and Women

**DOI:** 10.4172/2155-6113.s1-008

**Published:** 2012-06-03

**Authors:** Kimberly A. Kisler, John K. Williams

**Affiliations:** Department of Psychiatry & Biobehavioral Sciences, Semel Institute for Neuroscience & Human Behavior, University of California, Los Angeles, CA, USA

## Abstract

**Background:**

HIV prevention has rarely explored the impact of childhood sexual abuse (CSA) across health domains among African American men who have sex with men and women (MSMW). Early sexual experiences may influence perceptions of gender roles, sexual identity, and risks for HIV/AIDS. The attribute of masculinity is commonly associated with strength and success. However, a legacy of racism and oppression may pose challenges for African American men in achieving gender-based milestones. Instead, proxies for success may include masculinity constructs with hypersexual posturing and prowess that contradict sexual health messages.

**Methods:**

Two groups, each meeting twice for 90-minutes, of HIV-positive African American MSMW participated in discussions focusing on masculinity and sexual experiences. Participants were bisexual HIV-positive African American men who engaged in unprotected sex and had histories of CSA. Discussions were recorded, transcribed, and analyzed using consensual qualitative research and a constant comparison qualitative method.

**Results:**

Participant mean age was 40.5 years (n=16). Majority had a high school education (69%), half were unemployed, and almost two-thirds earned less than $20,000 annually. Three themes, each with two subthemes, emerged that described the sociocultural context for engaging in high-risk sexual behaviors, and included: 1) the importance of inhabiting a “traditional” masculine gender role with: a) general and b) sexual masculine traits; 2) the influence of conceptions of masculinity on sexual identity with the associations: a) between being gay and being effeminate and b) between being gay and being HIV-positive, and; 3) CSA experiences with: a) appraisal of CSA and b) early sexual experiences as rites of passage.

**Conclusion:**

Attempts to be masculine may contribute to high-risk sexual behaviors. Research needs to explore how early sexual experiences shape perceptions of masculinity and masculinity's influence on receiving health messages for African American MSMW who may prioritize a specific image over sexual risk reduction

## Introduction

Despite advances in prevention, detection, and treatment over the past thirty years, rates of HIV/AIDS continue to disproportionately impact distinct populations within the United States (U.S.). This disparity is especially salient among racial/ethnic minority groups, with African Americans being the most affected [1]. According to the Centers for Disease Control and Prevention (CDC), in 2009, African Americans represented 14% of the U.S. population but accounted for 44% of all new HIV infections [1]. African Americans, as compared to other racial/ethnic groups, also had the greatest number of cases at all stages of HIV infection [1]. In terms of mortality, HIV served as the ninth leading cause of death for all blacks, and the third leading cause of death for black men and women, 35–44 years of age in 2007 [1]. (Note: The terms African American and black are used interchangeably in an attempt to accurately reflect the language presented in the literature cited.)

The disparity in HIV/AIDS is magnified among African American men who have sex with men (MSM), where rates are substantially higher than within the general African American population [2,3]. According to the CDC, African American MSM are the most severely affected by HIV/AIDS in comparison to other high-risk groups such as injection drug users, high-risk heterosexuals, and white and Latino MSM [4]. Further, prevalence and incidence rates of HIV among black MSM rival even those in the developing world [5,6].

The largest HIV transmission category among blacks in general is sex with men, which in 2009 accounted for three-quarters of the new cases of HIV among black men and more than three-quarters of the cases among black women [1]. African American men who have sex with men and women (MSMW) are a high-risk population, serving as a source of HIV transmission to both men and women. However, little research has been conducted specifically with African American MSMW, a racial/ethnic and sexual minority group. Factors such as childhood sexual abuse, gender roles, and perceptions of masculinity have rarely been studied when examining risks for HIV despite the potential of having a significant impact on adult sexual behaviors.

## Childhood Sexual Abuse (CSA) and Sexual Risk Behaviors

In order to address the HIV epidemic, innovative research efforts must explore rarely examined factors that may influence sexual risk behaviors [7]. One important variable, experiences of childhood sexual abuse (CSA), has commonly been neglected within HIV prevention despite several studies having linked a history of CSA to a number of sexual health outcomes. Research on CSA among males has found that in comparison to nonabused men, abused men were more likely to engage in high-risk sexual behaviors, have more lifetime sexual partners, use condoms less frequently, have higher rates of sexually transmitted diseases, and have up to a two-fold increase in the rate of HIV [8,9]. More specifically, gay and bisexual men with a history of CSA were more likely to report unprotected anal intercourse, more sexual partners and events, and more sexual episodes under the influence of drugs, than their nonabused counterparts [10–12]. The implications of CSA being correlated with sexual health risks is especially significant, as rates of CSA among samples of MSM have been reported to be between 20% and 39.7% [11,13,14], with a significant rate of 16% found among a large national study with men [15]. In addition, among a sample of 456 HIV-positive MSM, a CSA rate of 15% was reported [16].

## CSA, Gender Roles, and Sexual Identity

In addition to sexual risk behaviors, a history of CSA has been found to impact both gender role and sexual identity development [17]. Holmes and Slap reported in their literature review on CSA among males that gender role confusion was more prevalent among men who had been abused versus those who had not been abused [9]. Gender nonconformity in children has also been suggested as a risk indicator of abuses, such as CSA, and of posttraumatic stress disorder [18]. Furthermore, male victims of sexual abuse have indicated more sexual identity confusion and increased attempts to reassert masculinity through heightened expressions of toughness and aggression [19].

One study defined issues related to sexual identity development as one of the “sleeper effects” of CSA [20]. That is, consequences of CSA may be triggered later in childhood development as issues related to the abuse become more salient [20]. In a community sample of heterosexual, gay, lesbian, and bisexual individuals, CSA was found to be a significant predictor of non-heterosexual orientation and depression, accounting for 8.5% of the covariance in the statistically significant relationship between sexual orientation and depression [21]. Also, when examining the sexual partnering of adults who were physically or sexually abused or neglected as children, only the men with histories of CSA were significantly more likely than the other groups to report same-sex sexual partners [22]. While caution must be taken in interpreting these findings, as they do not support a causal relationship between CSA and gay/bisexual identities, they do support the need to further assess the impact of sexual abuse on sexual and gender identity formation.

Research has begun to examine the influence of gender roles on health outcomes among men [23–26]. Several studies have suggested that the multiple identities of black MSM play a significant role in understanding the HIV disparity among this population [27–29]. For instance, conceptions of racial/ethnic, gender, and sexual identities, both by the individual and the larger African American community, may impact sexual decision-making, especially when it comes to sexual partner selection and risk-taking behaviors. Importantly, these decisions and behaviors are made and acted upon largely within a population that has a high prevalence of HIV and sexually transmitted diseases. Though topics like these have been posed as areas for future research, little work has been done to actually examine them in-depth.

Researchers have pointed to perceptions of masculinity as a potentially important variable in understanding HIV risk behaviors for black MSM and MSMW [27,30–33]. Pleck, Sonenstein, and Ku found that among a sample of black, Latino, and white adolescents, those who endorsed more traditional ideas of masculinity were more likely to engage in sexual risk behaviors, such as having more sexual partners and engaging in inconsistent condom use [34]. Also, the extent to which African American men appraise early sexual experiences as abuse may influence future sexual behaviors and decision-making. Understanding the meaning of these experiences within the sociocultural context of being an African American gay or bisexual male may present cognitive conflicts. To acknowledge the experience could imply vulnerability and weakness, while minimizing, reframing, or denying the experience could be protective. In the latter case, men may purposely or unconsciously exhibit behaviors that could be viewed as hyper-masculine in an attempt to avoid any signs of frailty.

The purpose of this study was to explore how CSA influences perceptions of masculinity, gender roles, and sexual identity and how these constructs impact sexual decision-making and HIV risk behaviors among a sample of HIV-positive African American MSMW. Findings have implications for the development of future prevention and risk reduction interventions.

## Methods

In order to explore the constructs of masculinity, gender roles, and sexual identity among African American MSMW, two semi-structured focus groups were conducted, with each group meeting twice for 90 minutes. To recruit participants, fliers advertising for a research study focusing on physical, psychological, and sexual health and barriers to achieving a healthier lifestyle were posted in four diverse community-based organizations (CBOs), as well as distributed at community health events in Los Angeles County. One CBO provides a range of health, outreach, testing, and prevention services for indigent adults. The second operates residential drug treatment programs. The third operates a cultural center focusing on mental and physical health issues affecting African Americans. The fourth organization provides HIV testing and related support services including case management, assistance with housing, and mental health services. Potential participants were screened for eligibility in-person or over the phone and had to qualify on eight inclusion criterion. They had to be: 1) self-identifying black or African American; 2) English-speaking; 3) 18 years of age or older; 4) HIV-positive (verified through test results, medical records, antiretroviral medication prescriptions, etc.); 5) a sexually active male with both male and female partners in the previous 90 days; 6) a sexually active male with at least one incident of unprotected vaginal or anal sex in the previous 90 days; 7) nongay-identifying; and 8) a male with a history of CSA.

For this study, CSA was defined as having experienced, before the age of 18, any unwanted or forced sexual contact (ranging from touching and fondling to intercourse) and/or having sexual experiences with someone at least five years older. To screen for CSA, seven questions inquiring about sexual experiences prior to the age of 18 were used. This screener assessed for abuse using descriptions of experiences with language such as, “Before the age of 18, did anyone attempt to have intercourse with you against your will?” Use of the term “childhood sexual abuse” was not used in the screening since early sexual experiences may not have been appraised as abuse despite fulfilling the definition of CSA. This manner of screening has been used previously and has been found to be reliable [35].

An interview guide for the semi-structured focus groups was developed and included thirteen questions, which explored general and sexual health, as well as issues related to gender roles, sexual identity, and interpersonal relationships. The focus groups were recorded and transcribed verbatim. Data analysis was guided by consensual qualitative research and a constant-comparison method based in grounded theory [36,37]. First, a four-person team individually read the focus group transcriptions and identified themes, which were then summarized in a matrix format and circulated among the team members. Subsequently, the team met to discuss the major themes and identify common subthemes. Consensus of themes and subthemes were required prior to the coding of qualitative data (i.e., applying consistently to quotations across the transcripts). Atlas.ti™ software was used to aid in the organization and coding of data.

## Results

### Demographics

A total of 16 men participated in the two focus groups, with one group having seven and the other having nine men. Sample characteristics are highlighted in Table 1. The mean age of the participants was 40.5 years (range 24–56, SD = 9.46). A little more than two-thirds of the men had completed high school or some college. Half were unable to work and/or were unemployed while almost 38% were working either part-time or full-time. The sample was relatively low-income with 62.5% earning less than $20,000 per year. While the entry criteria required all participants to be nongay-identifying and behaviorally bisexual, on a brief post-focus group demographic survey, half the sample identified as gay and almost another third identified as same gender loving. Overall the sample included predominantly middle-aged, high school-educated, and financially vulnerable men.

Three major themes, each with two subthemes, were identified. The theme of masculine gender roles focused on the meaning of being an African American man, and included the subthemes of general masculine and sexual masculine traits. The influence of masculinity on sexual identity was the second theme and focused on the meaning of not being heterosexual, especially within the African American community, and included the subthemes of the association between being gay and being effeminate and the association between being gay and being HIV-positive. The third theme, CSA experiences, focused on individual interpretations of early sexual experiences, and included subthemes of appraisal of CSA and early sexual experiences as rites of passage.

### Masculine gender roles

#### General masculine traits

The men across both focus groups prescribed to a narrow range of ideas regarding what attributes an African American male should encompass. An emphasis on acting “masculine” was consistently described as being highly valued. In particular, masculinity was defined by expectations of acting “hard” and expressing a constricted range of emotions.

In regards to cultural and gender expectations of being an African American man, one participant stated:

To always be that hard rock exterior person. Don't show your feelings; don't let anybody see you cry. Don't let anybody see your feminine side.

Another participant shared the significance of concealing emotions for African American men and went on to underscore the consequences of what would happen if emotions were displayed:

In the black community, boys were always told that you're not supposed to show your feelings, you're not supposed to cry, you're always supposed to be hard. By your uncles, by your father, older brothers, stuff likes that and other men outside the family. You were always told that. That's where a lot of black men get confused, because they have all these feelings building up inside of them and yet they don't want to show these feelings because they feel like they're either gonna get beat up by their uncles, get a whooping by their father because they're showing these feelings, so they get confused in their minds.

In addition to having negative consequences, such as being the potential victim of abuse if emotions were shown, many participants also believed that exhibiting aggressive behaviors, including perpetrating violence towards others, was considered a valued masculine trait. One participant expressed this idea in the following quote:

[Violence] that's part of defining you as a man, whether you're black, white, straight, or gay, you have to have the threat of violence within you whether or not you would step up to it or not, you just can't be a pussy. You can't be soft like that and take it laying down. Anybody - you just don't take it. That defines to me, manhood.

#### Sexual masculine traits

Engaging in sexual activities also emerged as a common attribute of masculinity. Having sex was considered an important rite of passage into manhood, especially for African American boys. One participant believed that not only having sex, but also having sex at a young age with considerably older men, made him feel like a man.

It was like, for young men [who] started having sex at a young age…for myself, it makes me feel like a man, and the men I was having sex with, made me feel more mature.

The expectation to have sex with women, despite being sexually attracted to men, was also identified as a more masculine trait. One participant described this phenomenon with the following quote:

When I was growing up, I knew what I was back then [gay], even though I still had to have this [sex with a woman]…I had to go fuck this girl, you know.

Another participant echoed a similar point when he rhetorically expressed the following quote:

What is it to teach someone to be a man? You know what I'm saying, it's fucking as many girls as possible, is that being a man?

### Conceptions of masculinity on sexual identity

Expected gender roles of African American men and the emphasis on being “masculine” also impacted the mens' perceptions of sexual identity. The theme of sexual identity included the subthemes of the associations between being gay and being effeminate and between being gay and being HIV-positive.

#### The association between being gay and being effeminate

Being gay or engaging in same-sex sexual behavior was repeatedly characterized by acting feminine and “soft” and as being the antithesis of masculinity. Many of the participants, despite engaging in same-sex sexual behaviors, stressed that it was critical to not “act gay”. Displays of hyper-masculinity were considered particularly important when trying to buffer the potential consequences of being a sexual minority. One participant described this as follows:

You don't want people to look at you and go, `he's a faggot,' because you go every place that you want to go and because some people just don't accept that. You have to keep up that mask on society - you're masculine through and through until you get behind closed doors. And that's another [story] all together. But it's basic social perception…everybody has to be macho. Anybody have a little tint of femininity in them that's outwardly shown, they're put to the side.

Another participant stated that his sexual identity was strongly related to how others treated him.

Any social event, anything where you're going to be viewed, you want to be viewed as a straight man because you're gonna gets your respect. `What's up dog?' You know.

For many of the participants, being gay had implications for how they were perceived as men (i.e., masculine or straight versus effeminate or “faggot”) and on what and where behaviors were exhibited (i.e., in public versus “behind closed doors”).

#### The association between being gay and being HIV-positive

A desire not to appear gay or bisexual coupled with an intense need to fulfill a certain masculine role was specifically identified as a factor that impeded African American men from getting tested for HIV. One man acknowledged fears of being thought of as gay because he felt it was commonly associated with HIV/AIDS. That is, the stigma of HIV/AIDS being a “gay” disease was highly prevalent. Importantly, this perception of being gay and therefore being HIV-infected was identified as a barrier to African American men getting tested for HIV.

I do have a problem with our Afro-American men not wanting to go have these [HIV] tests done because we're so manly and want to think that if they do find out, somebody's going to think that they're gay. That's the first thing - they can go [and say] `you got HIV from playing around,' you know.

### CSA experiences

As required in the eligibility criteria, all of the participants had experienced CSA. The meaning of CSA, as a theme, included appraisal of CSA and early sexual experiences as rites of passage as subthemes.

#### Appraisal of CSA

Approximately two-thirds of the sample viewed their childhood sexual experiences negatively, while the remainder appraised it as less traumatic with some viewing it positively. Several of the men specifically expressed that their perceptions of gender roles and sexual identity were influenced by these early sexual experiences. While one participant acknowledged that having sex with his older uncles was “wrong,” he still believed that the sexual experiences helped him to define his gender role as a man by enhancing his masculinity.

And it just made me feel like a man being able to have sex, even though I knew those were my uncles. I know that's wrong.

One participant negatively appraised his early sexual experiences and attributed these experiences to his lack of sexual desire for women.

I was 9 years old and my [female] babysitter forced me to do things…I really believe that is the reason why I do not like women. I tried to be straight but I don't like the way it feels - I feel safer with men. A lot of men don't say things like this because we are supposed to be strong and make the woman feel safe. But what about us?

Similarly, another participant shared how he believed that early sexual experiences impact men's ability to have healthy relationships and that they also contribute to psychological health issues.

…And some of them who have guilt, they try to hold that guilt in from having had it [sexual abuse] happen to them. So they suppress it. Even married - a lot of them end up divorced because of whatever issues. They have issues [about their sexuality], they just don't talk about it [being abused], even with their friends who are heterosexual. They don't talk about what happened to them when they were molested. It's not anything they want known.

Experiences of CSA also contributed to many of the mens' confusion surrounding their sexual identity. In the following quote, the participant described how adults in established institutions were having sex with boys and how it lent to mixed feelings. That is, he questioned how these behaviors could be unacceptable or perceived as unhealthy experiences when trusted and respected men were engaging in such behaviors.

We'd go in there and find out they're having sex with the boys that are in these group homes and foster homes. So it's kind of mixed feelings about what is right. You go to church and you hear about this. That it's not right. But if you have all these people that's doing this, I guess whatever feels good or whatever you're comfortable with, that's what you go with. And I think I'm comfortable with a male.

One man appraised his early sexual experience as one that allowed him to explore his sexual interest in men despite acknowledging the experience as being abuse.

My first one was this guy down the street. I don't know how he thought I would do this, [sex with a man], but he did. But anyways…I knew I wanted it, I was already feeling that way. It really didn't matter if it was molestation or whatever because I knew what I wanted.

#### Early sexual experiences as rites of passage

For some participants, early sexual experiences, which were by definition CSA, were framed as being rites of passage into manhood. One participant, who negatively appraised his childhood sexual experiences with a male role model (i.e., Big Brother); shared that the perpetrator explained the experience as a way for a boy to become a man.

It was like my Big Brother… he used to do things with me like play ball and my mom really liked the idea of me having a Big Brother. But one day he took me in the garage and pulled the door down. He told me he was going to make me a man. That was it. I knew I couldn't tell my mom because she thought he was good for me.

## Discussion

Participants in this study placed significant importance on fulfilling a specific male gender role, with traditional ideals of masculinity characterizing what they thought an African American male should be. Social science and public health literature has characterized hegemonic or culturally sanctioned displays of traditional masculinity to include aggression, the rejection of “feminine” characteristics, exhibiting stoicism, having a preoccupation with sex and an increased sex drive, being an economic provider, and serving as a protector of the home and family [38–40]. Because of racism, discrimination, and a history of oppression, African American men may not have equal access to traditional characteristics that exemplify being a successful man, such as money, status, and power [41,42]. Therefore, it has been theorized that some African American men may attempt to address the issue of lacking these symbols of masculinity by accentuating those traits that are more under their control [43–45].

Consistent with the literature, participants in this study identified traits of masculinity to include acting “hard” and being aggressive, showing little emotion, and engaging in sexual acts, particularly with women. Majors and Billson have deemed this phenomenon among some African American men as exhibiting the “cool” pose, where these masculine characteristics are exaggerated in order to make up for a lack of equal access to other, harder-to-attain attributes and resources [43]. For men who are racial/ethnic and sexual minorities, institutional and structural barriers extend to all aspects of their lives and may have direct and indirect effects on their health [46].

In terms of sexual identity, the men in this study repeatedly stressed that in order for African American men to uphold the masculine gender role, they could not identify as “gay.” Similar to our findings, other empirical data suggest that there are distinct disadvantages to identifying as gay or bisexual for black men [32,43,47,48]. Some studies have reported that African American communities tend to have more negative views on same-sex relationships than their white counterparts. Denizet-Lewis described black MSM as being “products of a black culture that deems masculinity and fatherhood as a black man's primary responsibility – and homosexuality as a white man's perversion” [49]. Specific groups within the black community, such as low-income women and highly religious men, tend to be the most intolerant [50]. Another study found that the parents of black students at a midwestern university indicated more often than the parents of other racial/ethnic groups of students that homosexuality was perverse and unnatural [51]. Fear of ostracism by family, friends, and the church in African American communities remains a prevalent reason to not identify as gay or bisexual [52–56]. While these forms of social support may offer protection from the racial discrimination experienced in the broader community, they may require that individuals conform to heterosexist ideas and institutions [50].

Overarching negative attitudes toward homosexuality, coupled with expectations of heterosexuality and traditional masculinity, can further complicate gender roles and sexual identity among African American MSMW. Mays and colleagues stated that African American MSM were likely to experience rejection due to their double minority status [28]. That is, they may suffer racial discrimination within white heterosexual and gay communities and sexual discrimination in African American communities. Consequently, this double stigma may deplete their standing as “real” men, both because of their racial/ethnic composition and their sexual behavior.

The meaning of being gay or bisexual is complicated for African American MSMW and impacts intimate, familial, and communal interpersonal relationships. Even the image of being perceived as gay or bisexual affects their lives. Participants in this study repeatedly described how feminine, non-masculine traits were associated with perceptions of being gay and of being HIV-positive. By exhibiting traits associated with more traditional conceptions of masculinity, a buffer against the effects of being a sexual minority could be established and be protective.

Negative attitudes toward not being heterosexual in African American communities can have significant repercussions for the health and well-being of African American MSMW. These men are in a vulnerable position as they must deal with two layers of stigma, racism and homophobia. They may have to be selective in disclosing their sexual identities, as letting others know about their same-sex sexual behaviors could compromise their status in their community [56]. Displays of hyper-masculinity may be a way to buffer discrimination based on sexual identity within African American communities, as well as racial discrimination within the broader society [57]. African American MSMW, however, may be endangering their health by attempting to maintain this masculine persona. Sexual prowess (i.e., many partners, frequent sex, etc.) and the stigma of being considered gay or having HIV may place them at increased risk for becoming infected with and transmitting HIV, as well as not getting tested for HIV and other sexually transmitted diseases. Our qualitative findings provide some context to recent empirical data which shows that while black MSM are as likely as other racial/ethnic MSM to ever get tested for HIV, they test less frequently and are more likely to have an unrecognized HIV infection [5,29].

Although all men in the sample had a history of CSA and many of the men acknowledged that their early sexual experiences were abusive and/or inappropriate, some still believed that those sexual experiences enhanced their masculinity and helped them to become men [58]. These findings demonstrate the potential incongruence that may exist between the appraisal of the sexual experience as abuse and positive feelings about perceptions of masculinity and manhood. Importantly, images of masculinity were intimately embedded in sexual activity. Unfortunately, positive images of masculinity may not necessarily equate to healthy sexual decision-making and may actually lead to increased risky sexual risk behaviors.

As evidenced among these men, even early sexual experiences with women, commonly perceived to be desired by African American boys and men, could be negatively appraised and be associated with poor health outcomes. Research assessing how appraisal of sexual experiences, especially CSA, affects general, mental, and sexual health is greatly needed. Understanding the relationship between CSA and gender roles and perceptions of masculinity may be critical if interventions are to influence healthy sexual decision-making among at-risk populations. Social norms and media that endorse sexual activity, especially among boys, may be both explicitly and implicitly supporting abusive situations, with negative sequelae being minimized or ignored. At a societal level, there is a need to examine the contributions of sexual messages that reach and/or target children. Normalizing all sex, including what would be defined as abuse, may confuse children and place them at risk for not disclosing abuse, revictimization, and poor mental health. While stereotypes and myths exist around black sexuality, research examining sexual norms within African American communities is lacking.

Several limitations did exist in this study. First, the sample size was small and therefore findings are not generalizable. Second, while the men acknowledged sexual abuse by both male and female perpetrators, the qualitative data referenced predominantly experiences with men. Research needs to explore whether appraisal of sexual abuse varies when the perpetrator is male versus female. Lastly, many questions remain regarding how men frame their sexual abuse experiences. Research needs to explore what coping strategies and skills are utilized to deal with past experiences and assist in the prevention of being revictimized. Despite these limitations, this small qualitative study identified important themes that need to be explored in larger qualitative and quantitative studies.

In conclusion, interventions must break out of the “cookie-cutter” approach to HIV prevention and risk reduction and begin to address the unique needs of diverse populations, such as African American MSMW. Research needs to examine how victims of CSA appraise their sexual experiences and how these experiences and their meanings influence adult sexual behaviors. Stigma surrounding sexual abuse and same-sex behaviors may contribute to feelings of isolation and sustain the culture of keeping these experiences “secret.” The health implications of CSA need to be addressed within interventions that allow for emotional, physical, and sexual healing. Behaviors must be connected to emotional and cognitive processes and need to be contexualized within a temporal and ecological framework. That is, past and current experiences and feelings, as well as individual, familial, and communal scripts may influence current sexual behaviors and thus, should be addressed through a holistic approach in future interventions.

For the African American MSMW in this study, societal pressures to “be a real man” were identified as contributing to unhealthy sexual decisions. Future interventions need to be tailored to acknowledge the role larger societal schemas, like perceptions of masculinity, have on HIV risk behaviors. Inclusion of social learning theory and critical thinking exercises that encourage African American MSMW to explore the formation of personal identities within the sociocultural context of their lives and framed across the lifespan will better enable these men to understand who they are, the choices they make, and the behaviors they carry out. Within this conceptual model, stigmas that surround being a sexual minority and having been sexually abused could be contextualized and understood as factors influencing personal health behaviors. At a community level, attention to these factors and the ways in which prevention messages are developed and marketed are critical to address, as African American MSMW may not respond to those that do not resonate with their definitions of masculinity and health. Attention to both individual and community level factors is necessary if HIV risk reduction interventions with African American MSMW are to be effective.

## Figures and Tables

**Table 1:** Participant Demographics.

	n	%
**Education**		
High school and some college	11	68.75
College degree	3	18.75
Graduate degree	2	12.5
**Employment**		
Full-time	4	25
Part-time	2	12.5
Unable to Work/Unemployed	8	50
Retired	2	12.5
**Income (total annual household)**		
Less than $5,000	5	31.25
$5,000 – 9,999	1	6.25
$10,000 – 19,999	4	25
$20,000 – 29,999	2	12.5
$30,000 – 40,000	2	12.5
Greater than $40,000	2	12.5
**Sexual Identity Label**		
Heterosexual	0	0
Bisexual	3	18.75
Gay	8	50
Same gender loving (SGL)	5	31.25
Down-Low (DL) / Homosexual / Queer	0	0
